# [Retracted] LncRNA GAS6 antisense RNA 1 facilitates the tumorigenesis of clear cell renal cell carcinoma by regulating the AMP‑activated protein kinase/mTOR signaling pathway

**DOI:** 10.3892/ol.2024.14259

**Published:** 2024-01-29

**Authors:** Xiaoyun Guo, Hongjun Li, Mei Zhang, Rong Li

Oncol Lett 22: 727, 2021; DOI: 10.3892/ol.2021.12988

Following the publication of the above article, a concerned reader drew to the Editor's attention that the cell migration and invasion assay data featured in Figs. 2D and 3D, and the cell colony formation assay data in Fig. 2A, were strikingly similar to data appearing in different form in other articles written by different authors at different research institutes that had either already been published elsewhere prior to the submission of this paper to *Oncology Letters*, or were under consideration for publication at around the same time.

In view of the fact that certain of these data had already apparently been published prior to the submission of this article for publication, the Editor of *Oncology Letters* has decided that this paper should be retracted from the Journal. The authors were asked for an explanation to account for these concerns, but the Editorial Office did not receive a reply. The Editor apologizes to the readership for any inconvenience caused.

